# Papillary thyroid carcinoma with Hashimoto’s thyroiditis: impact and correlation

**DOI:** 10.3389/fendo.2025.1512417

**Published:** 2025-04-11

**Authors:** Shengpeng Yao, Hong Zhang

**Affiliations:** Department of Thyroid Surgery, The Second Hospital of Jilin University, Changchun, China

**Keywords:** cancer, papillary thyroid carcinoma, Hashimoto’s thyroiditis, autoimmune disease, thyroid

## Abstract

Thyroid cancer is a malignant tumor of the endocrine system. Papillary thyroid carcinoma (PTC) is the most common form of thyroid cancer and has a comparatively better prognosis. An autoimmune disease called Hashimoto’s thyroiditis (HT) affects the thyroid and can cause lymphocyte infiltration in the thyroid tissue as well as hypothyroidism, which is characterized by increased levels of a certain antibody. It is currently assumed that there is a connection between PTC and HT. HT may increase the incidence of PTC and improve its prognosis by regulating gene expression, participating in common signaling pathways, and creating a specific immune microenvironment. In this review, we summarized the relationship between HT and PTC as well as the effects of coexisting HT on PTC and the possible mechanisms, thereby providing new perspectives for future research.

## 1 Introduction

Thyroid cancer is the most common tumor in the endocrine system and has the highest five-year relative survival rate of all cancers (1). Malignant thyroid neoplasms can be classified into seven types based on molecular biology and pathological features: follicular thyroid carcinoma, papillary thyroid carcinoma, invasive encapsulated follicular variant papillary thyroid carcinoma, oncocytic thyroid carcinoma, differentiated high-grade thyroid carcinoma, poorly differentiated thyroid carcinoma and anaplastic thyroid carcinoma (2). Among them, PTC is the most common type and the prognosis of the different subtypes also varies as well (2).

HT is one of the most common autoimmune diseases and the leading cause of hypothyroidism and shares epidemiological features with thyroid tumors: the incidence is higher in women than in men (3, 4). HT is caused by T cells mistakenly attacking thyroid tissue, resulting in the lymphoplasmacytic infiltration (5). Its diagnosis is based on the symptoms of hypothyroidism and the monitoring of thyroid peroxidase antibody (TPOAb), ultrasound examination can also provide assistance in differential diagnosis (5).

The role of inflammation and the immune system in the development of cancer is a concept that has been widely discussed and accepted in recent years. By providing a range of bioactive molecules, inflammatory cells can contribute to the formation of the tumor microenvironment and thereby supporting cancer progression (6, 7). Chronic inflammation and autoimmune responses often precede the development of certain cancers, indicating a correlation between sustained immune processes and the development of tumors (6, 8).

As a type of chronic immune disease, HT exerts multiple influences on the progression of PTC, including increasing the risk of developing PTC and improving prognosis, reflecting the relationships between them on multiple fronts (9, 10). Thus, by contrasting PTC with and without HT, this review provides new theoretical and experimental insights for clinical treatment while summarizing the effects of HT on PTC and the correlation between them.

## 2 Hashimoto’s thyroiditis is one of the risk factors for papillary thyroid carcinoma

Patients suffering from HT have an increased risk of developing thyroid cancer, especially PTC, in contrast to other thyroid cancers. Some retrospective studies have suggested a stronger association between HT and PTC, with this association being even more pronounced in nodal HT (10–12). Because it is an immune-mediated disease of the thyroid, HT does not have a significant impact on the overall cancer incidence in affected individuals, but it can lead to a pro-inflammatory state, and the elevated levels of thyroid-stimulating hormone (TSH) may contribute to the development of PTC by affecting the TSH receptor, which may play a role in the development of cancer (12–14). In contrast to the higher incidence of PTC and HT in women, a clear pattern is emerging within the HT-affected population in which men are more likely to develop PTC (15). The current perspective suggests that the occurrence of thyroid cancer in male patients has a higher degree of invasiveness at the time of diagnosis (16).

The conclusions suggest that HT can be regarded as a risk factor for PTC and plays an important role in both the development and progression of PTC. However, in children, current research is inconclusive as to whether HT increases the risk of developing PTC, indicating the need for further prospective studies (17, 18). It is also recommended to pay more attention to thyroid nodules found during ultrasound examination, especially in people with HT (19).

## 3 Hashimoto’s thyroiditis contributes to improving the prognosis of papillary thyroid carcinoma

Because it is a form of differentiated thyroid cancer, the treatment of choice for PTC is surgery. The prognosis is significantly better compared to other malignancies and the 5-year survival rate is more positive (20, 21). In the current consensus, PTC is prone to lymph node metastasis(LNM) (22). Because PTC has a generally good prognosis, there is no discernible relationship between the presence of HT in PTC and overall survival, nor is there a significant effect of LNM on the overall survival rate (23, 24). Therefore, the usefulness of overall survival as an indicator is not very important when we talk about how HT affects the prognosis of PTC.

The metastasis of PTC to lymph nodes is influenced by a variety of factors. As for central lymph node metastasis (CLNM), well-established factors such as male gender, tumor diameter over 1 cm, age, etc. are known, but the influence of HT on CLNM in PTC is still largely under debate (24, 25). Given that the BRAFV600E mutation is known to be a risk factor for LNM and that HT is negatively correlated with both the aggressiveness and metastasis of PTC and the BRAF mutation rate, it is reasonable to conclude that HT lowers the risk of LNM (25–29). Multifocal papillary thyroid carcinoma (MPTC) is also covered by this result (30).

However, some studies suggest that the coexistence of PTC and HT may not have a beneficial effect on LNM and may even act as a separate risk factor for LNM as opposed to a protective factor (14, 30, 31). These results appear to contradict previous conclusions.

The existence of these contradictory results may be attributed to a variety of factors. This contradictory situation may be partially explained by a more refined categorization and discussion of age groups and tumor sizes, as the implications of having HT differ for younger and older populations, and patients with HT are often diagnosed at a younger age (31–33).

Recent studies have shown that among other markers associated with the prognosis of PTC, patients with coexisting HT may have a lower incidence of vascular invasion, a longer recurrence-free survival period, a lower mortality and recurrence rate, and fewer cases of extrathyroidal extension (9, 34–37).

Nevertheless, it should be emphasized that there is still a great of disagreement about how HT affects neural invasion as well as the multifocality and bilaterality of PTC (9, 34, 38). Further investigation is required to substantiate certain conclusions that are not sufficiently supported by the available data.

Given the various factors discussed above, the general conclusion can be drawn that HT is a favorable factor for the prognosis of PTC, meaning that the prognosis is generally favorable for PTC patients who also suffer from HT, despite some controversy and different findings is better from various studies on the influence of HT on certain prognostic indicators of PTC (39–41).

## 4 The possible mechanisms and causes

Reviewing the previous results, it is clear that HT may have a dual impact on PTC. It can increase the likelihood of PTC occurring, but when present alongside PTC, it can also improve its prognosis. The underlying reasons for this complex influence appear to be due to various factors ranging from genetic elements to the immune system. BRAF belongs to the RAF kinase family and is involved in the Ras/Raf/MEK/ERK signaling pathway, which is associated with cell proliferation and growth (42). Thyroid cancer frequently has BRAF mutations, and the most common mutation found in PTC is the BRAFVal600Glu substitution (BRAFV600E) (26).

This mutation is not uncommon in PTC in both adults and children and has high specificity (43). Certain morphological features can specifically identify the presence of the BRAFV600E mutation in PTC (44). Moreover, traits linked to BRAF mutations include hypoechogenicity, irregular margins, and micro/macrocalcifications seen on ultrasound (45). Previous studies suggest that the overexpression of NIBAN1 and miRNA-222-3p in PTC may result from the BRAFV600E mutation, which in turn may facilitate PTC metastases (46, 47). It can also reduce the primary cilia to some extent, thereby promoting the invasion of PTC (48). Accordingly, it is thought to be related to the aggressiveness of thyroid cancer and includes features such as vascular invasion, LNM and extrathyroidal extension (26, 49, 50).

The frequency of BRAFV600E mutations is significantly lower in PTC cases associated with HT, and even in cases where BRAF mutations occur, HT can partially neutralize this effect (51–54). This could potentially be the reason why PTC patients with HT have a better prognosis.

BRAFV600E can lead to differential expression of specific long noncoding RNAs (lncRNAs), which are predominantly involved in calcium signaling pathway, MAPK signaling pathway and other signaling pathways (55). Additionally, the expression of certain lncRNAs has differential patterns in PTC and papillary thyroid carcinoma with Hashimoto’s thyroiditis (PTC-HT). For example, the expression of BRAF-activated non-coding RNA (BANCR), which is involved in the proliferation and invasion signaling pathways of PTC, was found to be decreased in PTC, moderately increased in HT, and significantly decreased in PTC-HT (56, 57). This dual effect may, to some extent, account for the consequence of a higher incidence of PTC in patients with HT.

The RET proto-oncogene and its rearranged form, the RET/PTC, are found in both PTC and HT, with a prevalence of approximately 20% in PTC, and HT is significantly associated with it. Initially considered specific for PTC diagnosis, RET/PTC is now identified in some benign disease like HT as detection sensitivity improves (58, 59).This process arises from chromosomal rearrangements that affect the MAPK signaling pathway and lead to its sustained activation (60). The most common rearrangements are RET/PTC-1 and RET/PTC-3 (61). Among them, activation of the most frequently observed RET/PTC1 can lead to the downregulation of immune checkpoints and thus play a role in tumor progression (62). It is important to note that patients with benign nodules who test positive for RET/PTC may experience faster growth rates (63). Whether RET/PTC can be considered an early event in the development of malignant tumors remains to be confirmed, current research on RET/PTC in HT is limited and controversial, warranting further investigation (64, 65). In summary, there is insufficient evidence to conclude that detecting RET/PTC mutations in the context of HT indicates the presence of malignant tumors. Moreover, its prognostic impact in PTC when present alone is limited (66).

In targeted therapy for PTC, experiments using organoid models have demonstrated that combining BRAF inhibitors, such as vemurafenib and dabrafenib, with inhibitors of MEK, RTK, CDK, and other targets produces better results, inhibiting cell proliferation while modulating the immune response (67, 68).

Additionally, although PTC is classified as a kind of cancer and HT is classified as an autoimmune disease, there is significant overlap in the genetic factors associated with both. Among them, ADH1B, ABR, SERPINA1, and LPAR5 have been identified as key genes, with EGR1 serving as a common transcription factor for SERPINA1, ABR and LPAR5 (69). Furthermore, HIF-1α and PD-L1 are important upstream regulatory factors in this process (70).

**Figure 1:**
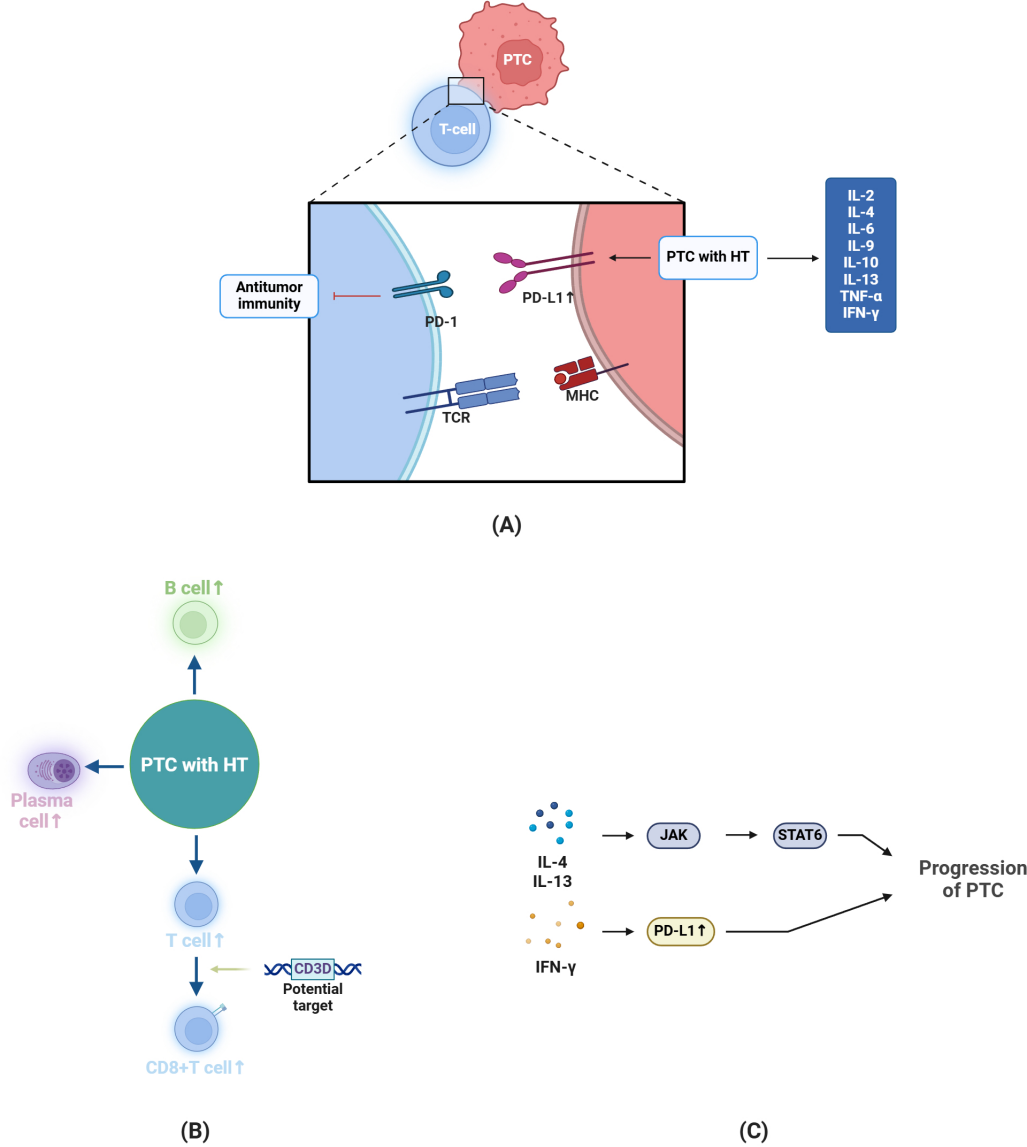
Changes and Potential Mechanisms in PTC-HT. **(A)** In antitumor immunity, T cells function via TCR binding to MHC molecules. In PTC patients with HT, the expression of PD-L1 is more pronounced, promoting tumor progression. Moreover, compared to PTC without HT, cytokines like IL-2 are upregulated. **(B)** In the case of PTC-HT, the number of B cells, plasma cells and T cells will increase, among which CD3D is a potential target to enhance the activation and **(C)** IL-4 and IL-13 can promote tumor progression through JAK-STAT pathway, and IFN-γ can promote PD-L1 expression in PTC cells to induce immune escape. The figure in this review is created by BioRender.com.

In terms of gene expression, the changes in PTC-HT are more pronounced than in HT alone, indicating a certain association between HT and the progression of PTC (69, 70). Among them, PD-L1, as a tumor marker associated with the malignancy of PTC, is positive in some PTC tissues, and its expression is even more pronounced in the context of HT (71–73). This might occur because of the various cytokines that HT, being a chronic inflammatory condition, produces and its subsequent effects on the tumor microenvironment (74). The microenvironment formed by HT can inhibit immune responses via the PD-1/PD-L1 pathway, and in tumor immunology, PD-L1 aids in facilitating the process of immune evasion (75). In the absence of PD-L1, T cells show enhanced antitumor activity (76) (**Figure 1A**).

Additionally, HT exhibits upregulated lactotransferrin (LTF) and CCL21 expression compared to PTC, while PTC-HT has higher expression of SERPINA1 and DMBT1 (77, 78). LTF, an immune-related factor, is associated with tumor invasion and growth when downregulated, CCL21 is related to the infiltration of lymphocytes into tissues, SERPINA1 is involved in multiple pathways in PTC progression and its high expression can inhibit the roles of KRAS and TNF-α in their signaling pathways via the NF - κB pathway, while highly - expressed DMBT1 can also suppress PTC through immune pathways (77–80). In conclusion, the distinct levels of factor expression between the two conditions provide an explanation for the improved prognosis in PTC-HT compared to PTC alone.

Since it is an autoimmune disease, the role of specific antibodies in the progression of PTC is essential and should not be overlooked. In HT, activation of cellular and humoral immunity to certain autoantigens may have effects on thyroid endocrine function (81). TPOAb and thyroglobulin antibody (TgAb) are both autoantibodies against the thyroid, with TPOAb being a diagnostic hallmark for HT (5). In patients with coexisting HT and PTC, elevated TPOAb levels (>1300 IU/mL) correlate with the multifocality of PTC, and preoperative TPOAb and TgAb levels may, to some extent, predict the risk of recurrence in these patients (82–84).

In patients with HT, TPOAb acts as a protective factor, whereas TgAb tends to be a risk factor that promotes the progression of PTC (85). Studies have indicated that high levels of TgAb IgG4 are a risk factor for the development of PTC, which is related to differences in epitopes (86).

Increased levels of TPOAb and TgAb can lead to elevated TSH expression, which subsequently stimulates the secretion of VEGF, and patients with HT who have higher levels of TSH are more susceptible to developing PTC, because TSH, by acting on the TSH receptor, can to some degree facilitate the progression of PTC (85–88). TSH can reduce the expression of p53 and E-Cadherin, counteract the cellular senescence induced by the BRAFV600E mutation, thereby promoting the progression of the tumor (89). This could partially explain the influence of elevated TSH levels on the increased incidence of PTC. Overall, the different levels of specific antibodies in PTC-HT patients may influence the prognosis of patients with PTC (90).

In terms of metabolism, PTC shares similar metabolic pathways regardless of the presence of HT (91). However, in PTC-HT, there is a significant increase in serum concentrations of glutamate and lysine, while the concentration of alanine decreases in comparison, which correlates negatively with TPOAb levels (91, 92).

The significance of the tumor microenvironment in cancer progression should not be overlooked, as it can confer tumors with signature characteristics at an earlier stage, which is particularly the case in the progression of PTC. Research has confirmed that the presence of HT can alleviate oxidative stress caused by PTC (7).

By promoting angiogenesis, VEGF supplies the tumor with nutrients (93). Compared to patients with PTC alone, those with PTC-HT exhibit lower expression levels of VEGF (94). In thyroid tissue, E-cadherin and its activator TGF-β1 are more highly expressed in patients with PTC complicated by HT, while the expression of N-cadherin and ICAM-1 is reduced (95). The two play different roles: N-cadherin tends to promote tumor cell metastasis, while E-cadherin can normally inhibit tumor invasion and its loss is associated with the epithelial-mesenchymal transition (EMT) (95, 96). Therefore, it can be concluded that HT generally exerts an inhibitory effect on the invasiveness of PTC.

The immune system can monitor and attack tumor cells, which is one of its functions. After cancer cells emerge, the immune system will try to attack and eliminate them. In some tumors, T - cell infiltration predicts a better prognosis. As a type of T cell, cytotoxic T lymphocytes (CTLs) can identify and specifically kill tumor cells after accepting antigens (97). This process relies on MHC I. regulatory T cells (Tregs), whose main function is immune suppression and which are often associated with a poor prognosis in tumor immunity, are also a current direction of immunotherapy by specifically targeting the tumor cell microenvironment (98, 99) (**Figure 1A**).

In terms of immunity, one of the characteristics of cancer is to evade the immune environment, and specific surface molecules under immune response promote the progression of the tumor (85). HT plays a significant role in the immune evasion mechanisms of PTC, which is also related to its impact on the tumor microenvironment. Patients with HT show a decrease in Tregs, which are involved in immune homeostasis regulation, and an increase in the CD4 to CD8 ratio (4). In addition, the thyroid is infiltrated by lymphocytes, predominantly T cells (4). Compared with other non-autoimmune thyroiditis, the number of CD8+ T cells in HT is significantly increased, which is associated with cytotoxic effects that lead to follicular destruction (100).The majority of B cells are located in the thyroid tissue and there are no significant fluctuations in the amount of circulating B cells (101).

In PTC-HT, as compared to PTC alone, there is an increased presence of CD3+, CD4+, CD8+ cells, B lymphocytes, and plasma cells in the thyroid tissue. As already mentioned, the density of CD4+ cells is higher than that of CD8+ cells (102–104). A higher number of CD8+ T cells is positively correlated with the disease-free survival of PTC patients (105). CD3D is a target in signal transduction that enhances the effector function of CD8+ T cells. Through an increase in CD8+ cell count, HT may have an impact on the activation of STAT6 and the tumor microenvironment (105, 106) (**Figures 1B, C**).

Compared to PTC alone, patients with HT have increased production of IL-2, IL-4, IL-6, IL-9, IL-10, IL-13, TNF-α, and IFN-γ, and there is also an increase in the expression of MHC I (related to IL-2 and IL-10) (107–110). While IL-6 helps thyroid cancer acquire EMT and stem cell-like properties, increased levels of IL-4 and IL-10 can also encourage the expression of anti-apoptotic proteins (111) (**Figures 1A, C**).

The different levels of immune cells and cytokines can explain why PTC with HT may have a better prognosis. In PTC with HT, T cells, especially CTLs and Tregs, dominate the tumor microenvironment (112). CD8+ T cell immunity against tumors relies on MHC I, whose absence can lead to immune evasion. HT is associated with increased IL-2 expression, which boosts MHC I expression, creating a significant difference in MHC I levels between PTC and PTC-HT (110). However, there’s insufficient research to draw definitive conclusions about B lymphocytes in this context (113).

In addition, it is noteworthy that HT is also strongly associated with other thyroid malignancies, including medullary thyroid carcinoma(MTC) and thyroid lymphoma(TL), but appears to be weakly associated with undifferentiated thyroid cancer and follicular thyroid carcinoma (114).

Although the relationship between HT and MTC remains controversial, a study has shown that their association is more significant when gender is considered (11, 115). HT may affect serum calcitonin (sCt) levels but doesn’t negate the strong suspicion of MTC when sCt is high (116, 117). Similar to other thyroid diseases, ultrasound can provide diagnostic value for MTC in patients with HT (118).

TL is a very rare disease in clinical practice, and it is usually manifested by rapid enlargement of neck masses and even compression of airway (119, 120). As an autoimmune disease, HT is associated with a higher risk of TL, and they both exhibit clonal bands with sequence similarity (121, 122). In some cases, primary TL has similar characteristics to HT under ultrasound and should be identified (123).

## 5 Impact of coexistent Hashimoto’s thyroiditis on papillary thyroid carcinoma diagnosis and treatment

Pathologically, PTC typically exhibits a papillary architecture and a propensity for lymphatic metastasis (22). HT presents with focal lesions, and its immunohistochemical characteristics are similar to those of PTC (124). PTC associated with HT tends to show tumorous fibrosis and anastomosing pseudovascular spaces on pathological examination (125).

In aspects of ultrasound and fine needle aspiration biopsy (FNAB), patients with PTC-HT have a lower proportion of large areas of calcification and psammoma bodies but a higher frequency of dense calcifications compared to PTC without HT (126). However, overall, the coexistence of HT does not significantly impact the outcomes of preoperative ultrasonography and FNAB (127). Furthermore, applying ultrasound findings and serological markers to deep learning may aid in the future diagnosis and detection of HT (128).

In patients with autoimmune thyroid diseases, the use of thyroid ultrasound examination helps in the early detection of PTC (19, 129). In ultrasonography, HT patients who demonstrate patterns of echogenic foci may be at an increased risk for PTC (130). Preoperative ultrasound examinations in PTC patients may be helpful in predicting the occurrence of CLNM (131). However, it is important to recognize that the presence of HT may complicate the interpretation of preoperative ultrasound findings related to CLNs in patients with PTC (132).

For thyroid cancer, surgery is the most common and traditional treatment method (22). When there is evidence of spread, a comprehensive clearance of the thyroid and any compromised tissues is attempted (22). In addition, treatment with radioactive iodine can also be carried out postoperatively in individual cases (22). Drawing from previous conclusions, patients with HT are at an elevated risk for the development of PTC, and thus, total thyroidectomy may be considered for them (133). With HT, thyroid surgery is more likely to encounter adhesions, and patients are at a higher risk of developing transient hypocalcemia postoperatively, which requires attention (134). Patients with PTC who have coexisting HT tend to respond better to radioactive iodine treatment (135). Nonetheless, to guarantee the efficacy of radioactive iodine therapy, it’s critical to modify the recommended dosage (135).

Endoscopic thyroidectomy is a relatively new surgical treatment method that does not increase the incidence of postoperative complications compared to open surgery and is considered a safe and reliable approach (136). The concomitant presence of HT may increase the duration of the procedure but does not have a significant impact on the results (137).

In PTC, common mutations include BRAFV600E and RET/PTC fusion. Given the favorable prognosis of PTC patients after surgery, targeted therapy is considered for those with potential poor prognosis, such as coexistence of RET/PTC and TERT promoter mutations (66). Sunitinib (mainly inhibits RET/PTC1 and RET/PTC3) and Sorafenib (inhibits RAF) in TKI have a certain effect for patients with advanced DTC or RAI-refractory DTC (138).

## 6 Discussion

In recent years, the incidence of thyroid tumors has increased, with papillary thyroid carcinoma being the most common form of thyroid cancer. It has distinct pathological features and indicators and both diagnostic and therapeutic approaches are well establishedclinically. The prognosis of PTC is one of the best compared to other types of cancer, making it one of the most well-researched diseases currently.

Hashimoto’s thyroiditis is an autoimmune disease caused by an abnormal immune system, which is the most common cause of primary hypothyroidism in non-iodine deficiency conditions. Patients may present with hyperthyroidism, hypothyroidism during the disease. The combination of clinical symptoms and the determination of hormones and antibodies in the blood helps to diagnose the disease, in which TPOAb is more specific and histological examination is not necessary.

Currently, numerous studies have demonstrated a link between PTC and HT. This article reviews the potential impacts of HT on PTC and the relationship between them, summarizing the latest research advancements from recent years.

HT exerts a dualistic effect on PTC. On the one hand, patients with HT as one of the risk factors for PTC are more susceptible to developing PTC. This susceptibility is associated with genetic changes, cytokine release, the inflammatory environment induced by HT, and higher TSH levels, all of which contribute significantly to shaping the tumor microenvironment. Additionally, HT and PTC share many common genes and similar metabolic pathways, which may determine their similarities in some signaling pathways, thus predisposing individuals to PTC. On the other hand, compared to patients without HT, those with PTC-HT often have a better prognosis. This is related to the different expression levels of factors such as E-cadherin and VEGF, as well as the presence of specific antibodies. Moreover, because HT is an autoimmune disease, immune cells in the tumor microenvironment of PTC will show significant differences in whether HT is accompanied or not. Because of the existence of these two effects, in the presence of HT, the diagnosis and treatment of PTC needs to be considered more circumstances, and specific diagnosis and treatment plans should be developed according to the different circumstances of individuals.

In the field of diagnosis and treatment, although the presence of HT has some impact on PTC, it does not affect the results of ultrasound and FNAB to such an extent that it would affect the assessment. Given the conclusion that HT is a risk factor for PTC, it is recommended to increase the use of ultrasound examinations in HT patients in clinical practice to detect the presence of PTC early and achieve better treatment outcomes. If PTC is confirmed and the patient also has HT, decisions regarding lymph node dissection should be made with greater caution. In addition, the dosage of radioactive iodine treatment should be adjusted to the specific conditions.

In the current research on HT and thyroid tumors, the association between HT and DTC (especially PTC) has been the most prominent, but other types of tumors (such as MTC with a poor prognosis) should still be concerned, although the probability of occurrence may be lower.

However, TL should be paid more attention to patients with HT. Although the occurrence probability is not high, once it occurs, it may be manifested as pressure on the trachea and esophagus, and its ultrasonic diagnosis and treatment plan are different from that of general thyroid tumors, so more care should be taken.

Unlike PTC, more cases are reported in the analysis discussing the association between HT and other malignancies, which may be related to the small number of cases, and the mechanism is not as fully explored as between PTC and HT. However, based on existing studies and discussions, no matter what kind of association, the role played by the chronic inflammatory characteristics of HT cannot be ignored.

Melanoma also has BRAF mutations, and clinical trials have been conducted to test the effects of BRAF inhibitors. In PTC, the presence of BRAF mutations often means a worse prognosis, and the combination of inhibitors targeting BRAF with multiple other inhibitors may lead to better outcomes.

RET/PTC is seen in a subset of PTC patients and was initially considered a specific feature, but it is now possible to detect this chromatin rearrangement in benign disease. There is still debate about this phenomenon: are there microscopic lesions that have not been detected by histology? It should be noted that even in benign lesions, the presence of RET/PTC may indicate a faster growth rate of nodules. Similarly, the corresponding inhibitors may have greater significance in patients with poor prognosis.

In the future, patients with HT should undergo more frequent follow-up, especially in groups more susceptible to developing PTC, such as individuals with BRAFV600E mutations or RET/PTC. The dual effects of HT on PTC require that treatment plans should consider the presence of HT to select the most effective therapeutic strategies. By focusing on the similarities between HT and PTC in the future, we can better explore the pathogenesis of PTC, thereby enabling more precise treatments.
